# Vaccine-Mediated Mechanisms Controlling *Francisella tularensis* SCHU S4 Growth in a Rat Co-Culture System

**DOI:** 10.3390/pathogens9050338

**Published:** 2020-04-30

**Authors:** Helena Lindgren, Kjell Eneslätt, Igor Golovliov, Carl Gelhaus, Patrik Rydén, Terry Wu, Anders Sjöstedt

**Affiliations:** 1 Department of Clinical Microbiology and Laboratory for Molecular Infection Medicine Sweden (MIMS), Umeå University, SE-901 85 Umeå, Sweden; helena.e.lindgren@umu.se (H.L.); kjell.eneslatt@umu.se (K.E.); igor.golovlev@umu.se (I.G.); 2 MRIGlobal, Kansas City, MO 64110, USA; cgelhaus@mriglobal.org; 3 Department of Mathematics and Mathematical Statistics, Umeå University, SE-901 85 Umeå, Sweden; patrik.ryden@umu.se; 4 Center for Infectious Disease & Immunity, Department of Internal Medicine, The University of New Mexico Health Science Center, Albuquerque, NM 87131, USA; TWu@salud.unm.edu

**Keywords:** *Francisella tularensis*, SCHU S4, in vitro co-culture model, rat immune response, correlates of protection, nitrite

## Abstract

*Francisella tularensis* causes the severe disease tularemia. In the present study, the aim was to identify correlates of protection in the rat co-culture model by investigating the immune responses using two vaccine candidates conferring distinct degrees of protection in rat and mouse models. The immune responses were characterized by use of splenocytes from naïve or Live vaccine strain- (LVS) or ∆*clpB/*∆*wbtC*-immunized Fischer 344 rats as effectors and bone marrow-derived macrophages infected with the highly virulent strain SCHU S4. A complex immune response was elicited, resulting in cytokine secretion, nitric oxide production, and efficient control of the intracellular bacterial growth. Addition of LVS-immune splenocytes elicited a significantly better control of bacterial growth than ∆*clpB/*∆*wbtC* splenocytes. This mirrored the efficacy of the vaccine candidates in the rat model. Lower levels of IFN-γ, TNF, fractalkine, IL-2, and nitrite were present in the co-cultures with ∆*clpB/*∆*wbtC* splenocytes than in those with splenocytes from LVS-immunized rats. Nitric oxide was found to be a correlate of protection, since the levels inversely correlated to the degree of protection and inhibition of nitric oxide production completely reversed the growth inhibition of SCHU S4. Overall, the results demonstrate that the co-culture assay with rat-derived cells is a suitable model to identify correlates of protection against highly virulent strains of *F. tularensis*

## 1. Introduction

The facultative intracellular bacterium, *Francisella tularensis*, is highly virulent and the etiological agent of tularemia, a potentially lethal disease in humans and many other mammals [1]. *F. tularensis* is classified as a Category A Select Agent, thereby being a potential biological weapon, since it can spread via aerosols, is extremely infectious, and cause disease with high morbidity. Natural outbreaks of tularemia may occur in many countries of the Northern hemisphere and are common and cause significant health problems in parts of Scandinavia, Eastern Europe, and Turkey, but rather uncommon elsewhere in the world. Subspecies *tularensis* (type A) and *holarctica* (type B) both cause human disease and although only the former may give rise to potentially lethal disease, also infection caused by subspecies *holarctica*, despite its lower virulence, may be serious. Currently, no licensed tularemia vaccine is available in the US or in Western Europe. The live vaccine strain of subsp. *holarctica* (LVS) has, however, been used to vaccinate laboratory personal in some Western countries. Its efficacy was demonstrated by the reduction of laboratory-acquired tularemia by 95% after its introduction [2]. 

Our previous studies of a set of mutants of *F. tularensis* SCHU S4 (type A) investigated their utility as live vaccines and it was observed that they demonstrated a spectrum of efficacies. In the mouse aerosol challenge model, some showed an efficacy at least as good as LVS, whereas some conferred intermediate, or poor protection [3,4,5]. Interestingly, the efficacy of the vaccine candidates in vivo was mirrored by the degree of control observed using a mouse in vitro co-culture system in which immune T-cells are added to macrophages infected with live bacteria [6]. A complex immune response was elicited in the co-cultures as demonstrated by T-cell activation, cytokine secretion, and nitric oxide production. Thus, the co-culture assay closely mimics the in vivo situation demonstrating the multiple interactions of several T cell subsets and with other types of immune cells. This makes it a suitable model to identify correlates of protection against *F. tularensis*. 

In contrast to mice, which are highly susceptible to virulent strains of *F. tularensis*, rats exhibit essentially similar susceptibility as humans and may therefore serve as a better model than the mouse [7]. In support of the utility of using rat cells in the co-culture model, it has been shown that peripheral blood leukocytes from immunized rats were able to control intra-macrophage growth of LVS in a co-culture assay and, as in the mouse model, it was demonstrated that the in vitro hierarchy of intra-macrophage growth control of LVS exhibited a pattern that directly correlated with the efficacy of the investigated vaccines to protect against challenge with a highly virulent strain in vivo [8]. Importantly, a similar co-culture assay was also developed using human-derived cells and this model successfully separated the efficacy of PBMC from LVS-vaccinated persons from non-vaccinated persons to control intracellular growth of the highly virulent strain SCHU S4 [9]. The degree of control correlated to T-cell activation and cytokine secretion. Identification of correlates of protection in the rat, mouse, and human co-culture models is of importance, since overlapping correlates can be used to predict the efficacy of a new vaccine candidate in humans.

IFN-γ has been identified as a correlate of protection for a number of intracellular infections and this is related to its critical role for activation of macrophages and the exquisite susceptibility of IFN-γ-deficient mice to the same infections. Although IFN-γ is central to the immune response against *Francisella*, IFN-γ per se is not sufficient to control the infection [10]. This was clearly demonstrated by use of Gbpchr3-KO mice, which despite high levels of circulating IFN-γ, were highly susceptible to *Francisella novicida* [10]. The presence of an IFN-γ-independent control of intracellular *F. tularensis* was further demonstrated by Elkins and co-workers in a co-culture assay [11]. 

In view of the severity of the disease and because outbreaks of tularemia are rare, there are obvious limitations regarding the possibility to perform human clinical trials to establish efficacy of a new vaccine, since there will be both ethical and practical limitations. Therefore, studies of a new vaccine against tularemia will be governed by the so called Animal Rule established by the US Food and Drug Administration, which states that a clinical trial to assess safety may be approved based on efficacy studies in animals [12], provided that a scientifically sound method has demonstrated that a vaccine candidate will provide the intended protection. The co-culture assay has the potential to meet this criterion, since it with high precision predicts the efficacy of vaccines in the mouse and rat models and is applicable to both human and animal cells. The assay has, however, not been approved by FDA and, before approval, the model therefore requires further characterization with regard to potential to predict efficacy of a vaccine in an animal model and the identification of correlates of protection. 

In the present study, the aim was to identify correlates of protection against SCHU S4 in the rat co-culture model by investigating the immune responses using two vaccine candidates conferring distinct degrees of protection against SCHU S4 in the in vivo rat model. After immunization of rats with LVS or ∆*clpB/*∆*wbtC*, a co-culture assay was established by use of splenocytes from immunized or naïve Fischer rats and BMDM infected with the SCHU S4 strain. The immune parameters measured, degree of control of SCHU S4 replication, levels of T-cell activation, and cytokine and nitrite secretion mirrored the efficacy of the vaccine candidates in vivo in the rat. Overall, the results demonstrate that the co-culture assay with rat-derived cells is a suitable model to identify correlates of protection against highly virulent strains of *F. tularensis.*

## 2. Results

### 2.1. Protective Ability of the Mutant Strains in the Rat Model

Rats were subcutaneously (SC) immunized with either LVS or Δ*clpB*/Δ*wbtC*, or injected with PBS, and 8 or 16 weeks thereafter challenged with aerosolized SCHU S4. In three separate experiments, with the challenge doses ranging from 2 × 10^3^ to 1 × 10^4^, a higher percentage of rats immunized with LVS survived compared to rats immunized with Δ*clpB*/Δ*wbtC* (Table 1) and all rats in the PBS-treated group died. In addition, there was a delayed time to death among the immunized rats who died relative to the rats in the PBS-treated group (*p* < 0.001; Table 1). Overall, the results demonstrate that rats immunized with LVS or Δ*clpB*/Δ*wbtC* were partly protected against a challenge with SCHU S4 and that LVS conferred more efficacious protection than Δ*clpB*/Δ*wbtC*.

### 2.2. Optimization of the Co-Culture Assay

**Figure 1:**
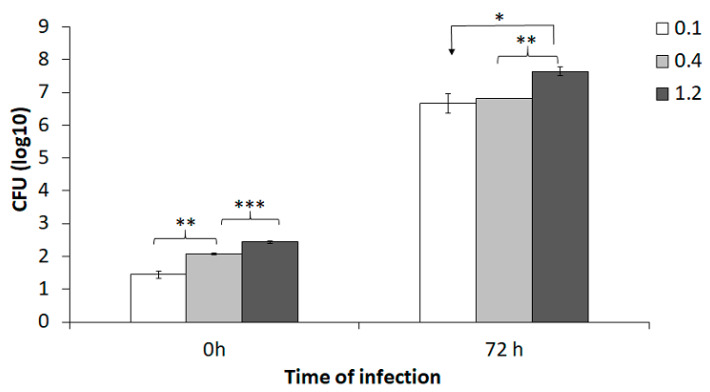
Effect of various multiplicity of infection (MOI) on the uptake and growth of SCHU S4 in BMDM. BMDM prepared from Fischer rats were seeded in a tissue culture plate and infected with an MOI (bacteria to BMDM) of 0.1, 0.4, or 1.2. Phagocytosis of the bacteria was allowed to proceed for 2 h after which the remaining extracellular bacteria were washed away and gentamicin added, designated as time 0 h. The number of colony forming units (CFU), were determined at 0 h and after 72 h of replication. The bars represent the means ± SEM of triplicate samples. * *p* < 0.05, ** *p* < 0.01, and *** *p* < 0.001. The data shown is from one representative experiment out of two.

To specifically evaluate the effect of various MOI (bacteria to BMDM) in the co-culture assay, BMDM were infected with an MOI of 0.1, 0.4, or 1.2 of the SCHU S4 strain and infection was followed for 72 h. The numbers of phagocytosed bacteria increased with increasing MOI (*p* < 0.01; Figure 1). After 72 h of incubation, the number of intracellular SCHU S4 was >6.0 log_10_ CFU and this represented >10,000-fold replication (Figure 1). At 72 h, there was significantly higher number of SCHU S4 in cultures infected with an MOI of 1.2 than in cultures infected with the lower MOIs (*p* < 0.05; Figure 1). 

**Figure 2:**
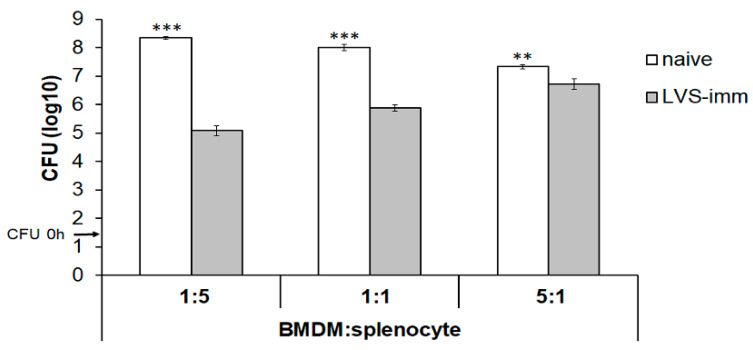
Optimization of the BMDM to splenocyte ratio in the co-culture assay. BMDM derived from Fischer rats were seeded at a density of 1 × 10^5^ cells/well in a 96-well plate. The monolayers of BMDM were infected with SCHU S4 at an MOI of 0.4 and the number of phagocytosed bacteria at start of the experiment was 1.5 log_10_. The infected monolayers were co-cultured with splenocytes prepared from naïve (white bars) or LVS-immunized (grey bars) Fischer rats at a BMDM:splenocyte ratio of 1:5, 1:1, or 5:1. The CFU were determined after 48 h of incubation. The bars represent the means ± SEM of triplicate samples. * *p* < 0.05, ** *p* < 0.01, and *** *p* < 0.001. The data shown is from one representative experiment out of two.

The second aim was to establish how the ratio of BMDM to splenocytes affected the growth of intracellular SCHU S4. To this end, rat BMDM monolayers were infected with SCHU S4 at an MOI of 0.4 and co-cultured with splenocytes from naïve or LVS-immunized rats at a BMDM to splenocyte ratio of 5:1, 1:1, or 1:5. With the ratio of 1:5, the number of SCHU S4 was >1000-fold lower in cultures with LVS-immune splenocytes than in cultures with naïve splenocytes (*p* < 0.001; Figure 2). This difference gradually decreased to a 100-fold and a less than 10-fold difference when the ratio was 1:1 or 5:1, respectively (Figure 2). Nitrite accumulation in the culture supernatant, which reflects nitric oxide production from iNOS, reached the highest concentrations in cultures infected with a BMDM:splenocyte ratio of 1:5 (data not shown). 

**Figure 3:**
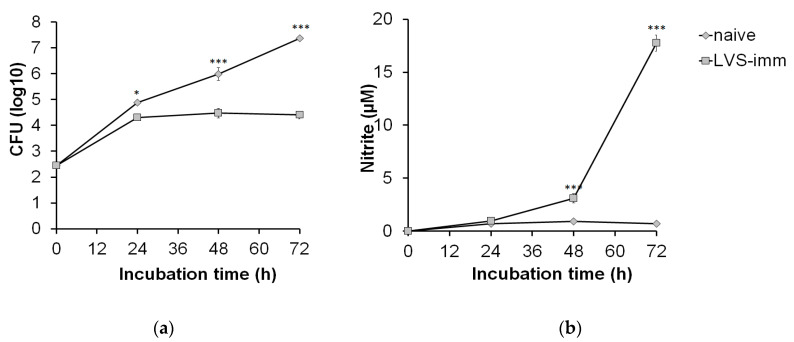
Kinetics of growth of SCHU S4 in BMDM and nitrite production in the co-culture assay. BMDM prepared from Fischer rats were seeded in a tissue culture plate and infected with a MOI of 0.4. The infected monolayers were co-cultured with splenocytes prepared from naïve or LVS-immunized Fischer rats at a BMDM:splenocyte ratio of 1:5. After 0, 24, 48, and 72 h of incubation, (**a**) the number of bacteria in the cultures was determined and (**b**) nitrite levels in the culture supernatant measured. The bars represent the means ± SEM of triplicate samples. * *p* < 0.05, ** *p* < 0.01, and *** *p* < 0.001. The data shown is from one representative experiment out of three.

Next, the kinetics of growth of SCHU S4 in rat BMDM and the growth inhibition of SCHU S4 induced by LVS-immune splenocytes were assessed. Based on the aforementioned results, an MOI of 0.4 and a ratio of BMDM to splenocytes of 1:5 were chosen. Until 24 h, SCHU S4 showed rapid growth in co-cultures regardless of which splenocyte population had been added (Figure 3a). However, between 24 and 72 h, there was no net growth of SCHU S4 in co-cultures with LVS-immune splenocytes, whereas in co-cultures with naïve splenocytes, SCHU S4 grew unrestrictedly, thereby reaching significantly higher numbers than in cultures with LVS-immune splenocytes at both the 48 and 72 h time points (*p* < 0.001; Figure 3a). The nitrite concentrations in co-cultures with LVS-immune splenocytes were below the detection limit at 24 h and reached approximately 3 µM at 48 h and were above 15 µM at 72 h (Figure 3b). In co-cultures with naïve splenocytes, the nitrite levels were below the detection limit regardless of time point.

Altogether, the experiments showed that co-cultures infected with an MOI of 0.4 and a BMDM to splenocyte ratio of 1:5 effectively controlled the SCHU S4 infection and led to significant nitric oxide production when immune splenocytes were added. This was apparent after both 48 and 72 h of infection. However, at 72 h SCHU S4 reached very high numbers in the cultures with naïve splenocytes, resulting in extensive cell lysis. Therefore, the 48 h time point was chosen for analysis in the subsequent experiments. 

### 2.3. Efficacy of Immune Splenocytes from Fischer Rats to Induce Protection Against SCHU S4 and the Role of Nitric Oxide in the Co-Culture Assay

**Figure 4:**
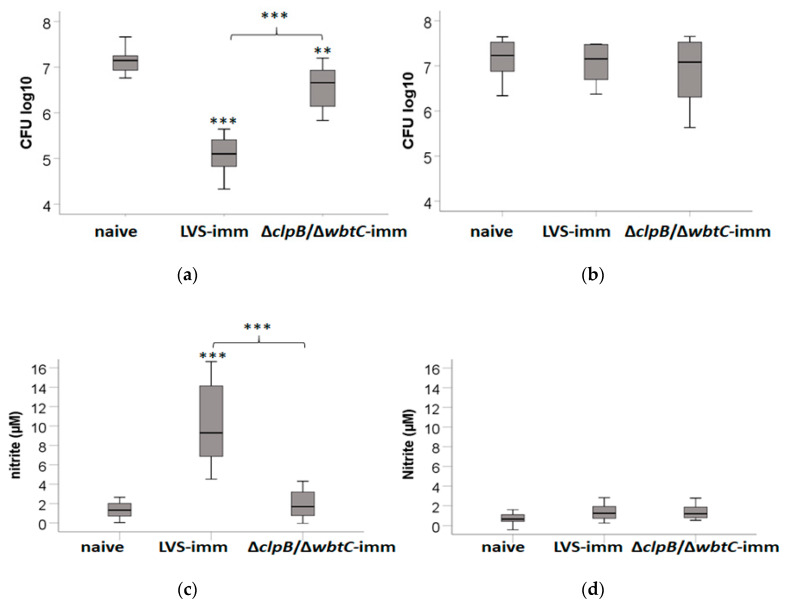
Control of the SCHU S4 infection and effect of NMMLA in the co-culture assay. SCHU S4-infected monolayers of BMDM were co-cultured with splenocytes from naïve or LVS- or ∆*clpB/*∆*wbtC*-immunized Fischer rats. After 48 h of incubation, the CFU was determined in cultures (**a**) without NMMLA and in cultures (**b**) with NMMLA. At the same time point, (**c**) nitrite levels in supernatants from cultures without NMMLA, or (**d**) with NMMLA were determined. At 0 h the BMDM monolayer contained 2.3 ± 0.1 CFU log_10_ of SCHU S4. Stars above the boxes indicate significant differences vs. cultures with naïve splenocytes and stars above the brackets indicate significant differences between the indicated groups. * *p* < 0.05, ** *p* < 0.01, and *** *p* < 0.001. The boxes summarize data from three cultures with NMMLA and five cultures without NMMLA, with triplicate samples in each.

The results from the rat model showed that LVS immunization induced a better protection than ∆*clpB/*∆*wbtC* against a challenge with SCHU S4 (Table 1). We then wanted to test if this vaccine efficacy could be reflected in the co-culture assay optimized herein. To this end, SCHU S4-infected monolayers of BMDM were co-cultured with splenocytes from naïve, LVS-, or ∆*clpB/*∆*wbtC*-immunized Fischer rats. At 0 h, the BMDM monolayer contained approximately 2.3 ± 0.1 CFU log_10_. By 48 h, the median bacterial numbers were 7.1 CFU log_10_ in co-cultures with naïve cells and this was 3-fold higher than in co-cultures with ∆*clpB/*∆*wbtC*-immune cells (*p* < 0.01) and about 100-fold higher than in co-cultures with LVS-immune cells (*p* < 0.001; Figure 4a). Notably, the ability of ∆*clpB/*∆*wbtC*-immune cells to contain the replication of SCHU S4 was much reduced compared to LVS-immune cells (*p* < 0.001; Figure 4a). Nitrite levels in supernatants from co-cultures with LVS-immune cells were higher than in supernatants from co-cultures with naïve cells or ∆*clpB*/∆*wbtC*-immune cells (*p* < 0.001; Figure 4c). Although nitrite levels in ∆*clpB/*∆*wbtC* cultures were slightly higher than in co-cultures with naïve cells, the difference was not significant (*p* > 0.05; Figure 4c).

Next, it was assessed if nitric oxide produced in the co-cultures contributed to the growth inhibition of SCHU S4. To this end, NMMLA, a competitive inhibitor of iNOS, which produces most of the nitric oxide, was used. Strikingly, addition of NMMLA abolished the growth inhibition conferred by the immune splenocytes and SCHU S4 grew as rapidly as in cultures with naïve splenocytes (Figure 4b). The levels of nitrite in cultures with NMMLA, or in co-cultures with naïve splenocytes were close to, or below the detection limit of 1 µM (Figure 4d). 

In summary, LVS-immune splenocytes elicited a significantly better control of SCHU S4 than ∆*clpB/*∆*wbtC*-immune splenocytes. The control of infection was inversely correlated to nitrite levels, since cultures with splenocytes from ∆*clpB/*∆*wbtC*-immunized rats contained lower levels than those with splenocytes from LVS-immunized rats. In addition, inhibition of iNOS abolished the control of infection otherwise conferred by LVS- or ∆*clpB/*∆*wbtC*-immune splenocytes. 

### 2.4. Efficacy of Splenocytes from Fischer Rats Immunized with LVS or ∆clpB/∆wbtC to Induce Cytokine Secretion in the Co-Culture Assay and Correlation of Cytokines to Nitrite

**Figure 5:**
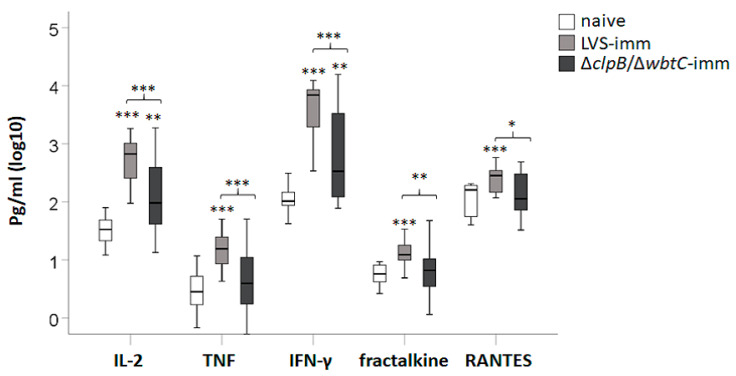
Cytokine accumulation in the supernatants during the co-culture assay. The supernatants were collected after 48 h of incubation and analyzed by Luminex. Stars above the boxes indicate significant differences vs. cultures with naïve splenocytes and stars above the brackets indicate significant differences between the indicated groups. * *p* < 0.05, ** *p* < 0.01, and *** *p* < 0.001. The boxes include data from 18–21 samples collected from up to nine separate experiments.

To assess the efficacy of splenocytes from immune Fischer rats to induce cytokine secretion in the co-culture assay, supernatants were collected after 48 h of infection and analyzed by Luminex. In total 27 cytokines were analyzed and the mean ± SEM for each group is presented in Supplementary Table S1. G-CSF, IL-5, IL-17, IP-10, IL-2, IFN-γ, TNF, fractalkine (CX3CL1), and RANTES concentrations were higher in co-cultures with LVS-immune splenocytes vs. co-cultures with naïve splenocytes (Table S1 and Figure 5; *p* < 0.05–0.001). In contrast, only levels of IL-2, IFN-γ, and GM-CSF were significantly higher in co-cultures with ∆*clpB/*∆*wbtC* vs. co-cultures with naïve splenocytes (Table S1 and Figure 5; *p* < 0.05–0.01). Notably, IL-2, TNF, IFN-γ, fractalkine, and RANTES were expressed at higher levels in co-cultures with LVS-immune splenocytes vs. co-cultures with ∆*clpB/*∆*wbtC*-immune splenocytes (Figure 5; *p* < 0.05–0.001). Moreover, IL-2, IFN-γ, TNF, fractalkine, and RANTES appeared to be co-regulated as their levels were highly correlated to each other; Spearman’s rho > 0.7 (*p* < 0.001; Table 2). 

NO production from iNOS was crucial to control SCHU S4 in the co-culture assay as demonstrated in Figure 4. Since expression of iNOS is induced in response to various cytokines, it was of relevance to investigate how the levels of the secreted cytokine in the co-cultures correlated to the nitrite levels. The cytokines giving the highest correlation coefficient, Spearman’s rho > 0.65, (*p* < 0.001) were IL-2, IFN-γ, fractalkine, TNF, and RANTES (Table 2). Notably, IL-2, IFN-γ, fractalkine, and TNF were secreted at lower levels in co-cultures with ∆*clpB/*∆*wbtC*-immune splenocytes than in co-cultures with LVS-immune splenocytes (Figure 5). Correlations were further analyzed for each group of co-cultures, i.e., co-cultures with naïve splenocytes, LVS-, or ∆*clpB/*∆*wbtC*-immune splenocytes. The correlation between nitrite and each of IFN-γ, IL-2, fractalkine, TNF, and RANTES was high in co-cultures with LVS-immune splenocytes (*p* < 0.01; Table 3). In contrast, for co-cultures with ∆*clpB/*∆*wbtC*-immune splenocytes, the correlation between IFN-γ and nitrite was not significant, whereas the significance was *p* < 0.05 for the other cytokines (Table 3). 

In summary, co-cultures with splenocytes from ∆*clpB*/∆*wbtC*-immunized rats contained lower concentrations of IL-2, TNF, IFN-γ, fractalkine, and RANTES than those with splenocytes from LVS-immunized rats and the levels of all of these cytokines were highly correlated to each other and to levels of nitrite. 

### 2.5. Analysis of IFN-γ, TNF-, and IL-17-Producing T-Cells Collected from the Co-Cultures

To determine the role of T-cells for cytokine production in the co-culture assays, splenocytes were collected from the wells after 48 h and analyzed by FACS for the presence of CD4 and CD8 T-cells expressing IFN-γ, TNF, or IL-17. 

**Figure 6:**
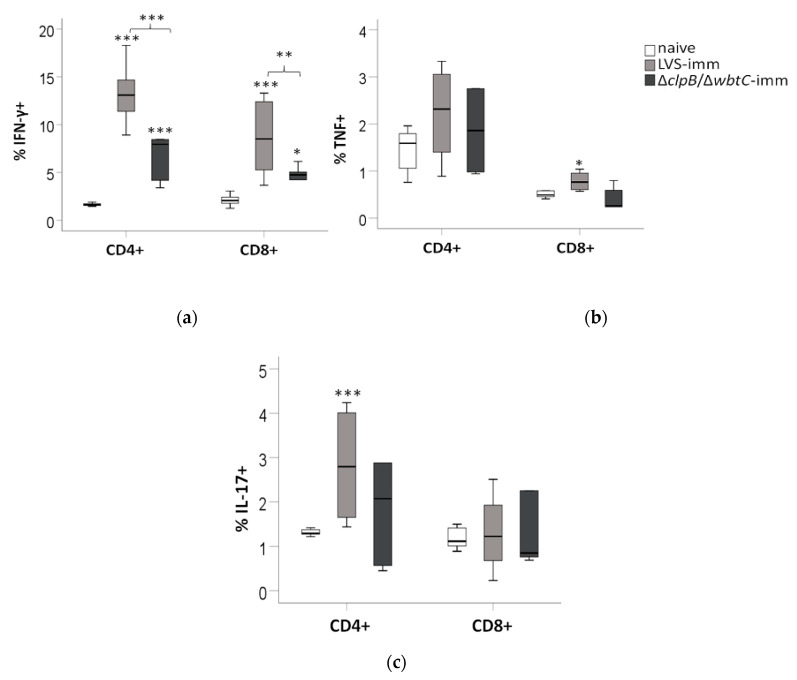
FACS analysis of cellular phenotypes derived from co-cultures after 48 h of incubation. The cells in the cultures were stained for the cell surface markers, CD3, CD4, and CD8, and for intracellular expression of (**a**) IFN-γ, (**b**) TNF, and (**c**) IL-17. Stars above the boxes indicate significant differences vs. naïve splenocytes and stars above the brackets indicate significant difference between the indicated groups. The boxes constitute values from eight separate samples generated in four different experiments.

The LVS-immune splenocyte population contained significantly higher percentages of CD4/IFN-γ-positive and CD8/IFN-γ-positive cells than the ∆*clpB/*∆*wbtC*-immune population (*p* < 0.001 and *p* < 0.01, respectively), which in turn contained a higher percentage than the naive population (*p* < 0.001 and *p* < 0.05, respectively; Figure 6a).

The percentage of CD4/TNF-positive cells was slightly higher in the LVS- and ∆*clpB/*∆*wbtC*-immune splenocyte populations than in the naïve cell population, although this was not significant and there were no significant differences among the two immune cell populations (Figure 6b). There was an increase in the percentage of the CD8/TNF-positive cells and CD4/IL-17-positive cells among the LVS-immune cell population (*p* < 0.05 and *p* < 0.10, respectively) vs. the naïve population, whereas this was not the case for the ∆*clpB/*∆*wbtC* population (Figure 6b,c). The percentage of CD8/IL-17-positive cells was similar among the groups (Figure 6c).

In summary, the FACS analysis revealed that the T-cell responses of ∆*clpB/*∆*wbtC*-immune cells to the *F. tularensis* antigen was less robust than those of LVS-immune cells, manifested as reduced populations of CD4 and CD8 T-cells expressing IFN-γ, but also fewer TNF- or IL-17-expressing T-cells.

### 2.6. Classification to Investigate the Multivariate Relations

We asked how well the variables CFU, nitrite, and the levels of the secreted cytokines IFN-γ, IL-2, TNF, fractalkine, RANTES G-CSF, IL-4, IL-12p70, and IL-17 could predict whether the cells had been derived from naïve, LVS-, or ∆*clpB/*∆*wbtC* vaccinated rats. Linear discriminate analysis was used to perform the classification and cross validation was used to estimate the model’s ability to predict the type of immunization. 

The best performance when only a single variable was included in the modeling was obtained for CFU with 69% correctly classified samples (Table 4). The performance increased substantially when two variables were included in the modeling. Here the best performing model, classifying 93.1% of the samples correctly, included the variables CFU and IFN-γ (Table 4). The overall best performing model included CFU, nitrite, IL-2, and TNF with an accuracy of 94.8% (Table 4). For this model only three samples were misclassified; one LVS-immunized rat was predicted to be immunized with ∆*clpB/*∆*wbtC* and two of the ∆*clpB/*∆*wbtC*-immunized rats were predicted to be naïve (Table 5). 

In summary, combining several of the variables CFU, nitrite, and secreted cytokines in an LDA modeling approach showed that the combination of CFU, nitrite, IL-2, and TNF efficiently discriminated between cultures with LVS splenocytes and cultures with naïve, or ∆*clpB/*∆*wbtC*-immune splenocytes. 

## 3. Discussion

Investigations of vaccine-mediated protective mechanisms against *F. tularensis* require relevant animal models and this is even more so for tularemia vaccines than for studies of other vaccines, since human efficacy studies are unlikely to be of sufficient power in view of the relative rarity of the disease. Even establishing relevant animal models for tularemia is challenging, since there are practical limitations, such as the availability of animal BSL3 facilities, the expertise required to perform precision aerosol experiments, or equipment required to assess the immunological responses in such BSL3 facilities. Therefore, co-culture models are of much interest since they circumvent the need for animal BSL3 models, but still allow for a very detailed immunological characterization of the vaccine-mediated responses. Such models will be important tools to evaluate potential vaccine candidates and to identify correlates of protection; the latter ones will be required to bridge between species and to obtain licensure based on the Animal Rule. An additional, complicating factor relates to the fact that cell-mediated immunity (CMI) is essential for protection against tularemia, but there is no validated assay for identification of cell-mediated correlates of protection. In fact, there is no validated assay to identify protective correlates against any intracellular pathogen, although many of them are important human pathogens. 

Co-culture assays have been extensively utilized to investigate vaccine-mediated mechanisms against tularemia in the mouse model, and there are also some studies that have utilized the assays based on rat and human cells [8,9]. The published studies demonstrate that co-culture assays are promising surrogate methods that could fulfill the requirements of the Animal Rule. Thus, the present study was aimed at investigating the usefulness of the rat co-culture model to evaluate protective mechanisms against the highly virulent SCHU S4 strain. There were several rationales for this; one of which is the fact that most of the studies performed with co-cultures have evaluated the immune responses using mouse cells against attenuated *F. tularensis* strains, such as the LVS strain. Therefore, there is a definite need to utilize co-culture assays based on the use of highly virulent *F. tularensis* strains and rat cells, since accumulating evidence has demonstrated the additional benefits of using the Fischer 344 rat model in comparison to any mouse model for tularemia [8]. 

In the present study, rats were immunized subcutaneously to mimic the human LVS vaccination, which is administered via scarification. Moreover, by using this route, the findings could directly be compared to previously published studies using the mouse co-culture model, since most often these studies have been based on subcutaneous vaccination. Importantly, to maximize the relevance of the information obtained, we utilized a highly virulent subspecies *tularensis* strain in the co-culture assay. We observed that the MOI and the BMDM to splenocyte ratio affected the control of infection. An additional important and practical finding was that the co-culture assay including splenocytes thawed from a frozen stock still discriminated the vaccine history of the cells with regard to their ability to induce control of intracellular SCHU S4, NO production, secretion of cytokines, and T-cell activation. The ability to prepare and store the splenocytes in liquid nitrogen saves considerable experimental time and greatly reduces the number of animals required to perform repeated experiments. 

By utilizing a vaccine strain conferring intermediate protection, ∆*clpB/*∆*wbtC*, it was possible to delineate which immune responses best correlated with protection, since the LVS strain confers efficacious protection in rats. Corroborating the in vivo findings, we observed that the control of bacterial replication mediated by LVS-immune splenocytes was superior to that conferred by ∆*clpB/*∆*wbtC*-immune splenocytes. Concomitantly, the better control mediated by the former strain strongly correlated to higher levels of secreted IFN-γ, TNF, fractalkine, IL-2, RANTES, and nitrite and a higher frequency of CD4^+^ or CD8^+^ T-cells expressing IFN-γ, TNF, and IL-17. Linear discriminate analysis further identified CFU, nitrite, IFN-γ, TNF, and IL-2 as correlates of protection, since these variables efficiently discriminated LVS-immunized rats from naïve or ∆*clpB/*∆*wbtC*-immunized rats. Additionally, in the mouse model, Th1-related cytokines, such as IFN-γ, TNF, and MCP-1, have been found to correlate to protection against SCHU S4 when vaccines conferring different degrees of protection were used [5]. We showed recently that addition of spleen cells from immune mice in a co-culture model resulted in high levels of nitric oxide, IL-17, IFN-γ, and GM-CSF and these levels strongly correlated to control of SCHU S4 infection in the model [6]. A recent study utilizing a co-culture model based on peripheral blood cells from LVS-immunized rats demonstrated that correlates could be identified that predicted protection against respiratory challenge of Fischer 344 rats with fully virulent *F*. *tularensis* [8]. The correlates included transcriptional upregulation of IFN-γ, IL-21, NOS2, LTA, T-bet, IL-12rβ2, and CCL5. The results from these previous studies utilizing mouse or rat cells show definite similarities with regard to the protective correlates identified in the present study. Altogether, the results support the notion that co-culture assays utilizing various types of mouse or rat cells are useful for identifying protective correlates against tularemia. 

We have also established a human assay based on the use of adherent cells as the source of infected macrophages and non-adherent peripheral blood mononuclear cells as effector cells, thereby resembling the rat and mouse co-culture assays [9]. In the human assay, secretion of IFN-γ, TNF, and MIP-1β correlated to control of infection. Thus, again, several Th1 cytokines were identified, corroborating their role for protective responses even against human tularemia. 

Importantly, our findings using the rat co-culture model demonstrate that the degree of protection conferred by the spleen cells closely correlate to the degree of protection observed in vivo in the rat model. We observed that immunization with the ∆*clpB/*∆*wbtC* strain conferred less potent protection than the LVS strain. This also demonstrates that the use of spleen cells as effectors in the co-culture assay seems to be a highly relevant source and closely mimics the ability to control systemic infection in mice and rats. 

We did not investigate the contribution of individual cytokines for the control of infection in the co-culture model, however, we did identify a critical role of NO. The inverse correlation between CFU and nitrite levels was very high, and the protective role of NO was corroborated, since inhibition of iNOS fully reversed the growth inhibition of SCHU S4. Besides this study, it has been observed that control of LVS and SCHU S4 infection in mouse and rat co-culture models correlated very well with levels of NO [6,8]. Importantly, nitrite levels were highly correlated to IFN-γ, TNF, fractalkine, IL-2, and RANTES, of which IFN-γ and TNF are potent inducers of iNOS transcription. Thus, the low levels of nitrite produced in the Δ*clpB*/Δ*wbtC* co-culture likely reflect the low levels of cytokines produced in this culture.

*F. novicida* is closely related to *F. tularensis* and the requirements for control of infection with the former bacterium has been carefully studied. Interestingly, the IFN-γ-mediated control is dependent on the guanylate-binding proteins GBP2 and GBP5, both of which are IFN-γ-inducible [13,14]. The critical role of the two proteins has also been validated for infection with the LVS strain, but they do not appear to contribute to control of the SCHU S4 strain [10]. Moreover, murine macrophages effectively control intracellular multiplication of *F. tularensis* LVS after IFN-γ activation and this is conferred by mechanisms involving both reactive nitrogen species, such as NO, and reactive oxygen species. However, the roles of these reactive species are less obvious when virulent strains are studied [15,16,17,18,19,20,21]. Collectively, the data from the animal models imply that the mechanisms for controlling infections with highly virulent strains are distinct from those controlling the attenuated LVS and *F. novicida* strains. Therefore, there is a need to include virulent strains in the co-culture models in order to identify the correlates of immunity and protection that presumably are clinically relevant.

Importantly, the present data demonstrate that effective control of infection with virulent *F. tularensis* bacteria is observed in the rat co-culture model and this demonstrates that it should be possible to identify similar human vaccine-mediated immune responses, thereby allowing bridging of studies among species as required by the Animal Rule. In turn, this could lead to the development of novel vaccines of general utility for protection against other strains of the highly virulent subspecies *tularensis*. Importantly, compared to the rat model, the mouse model that has been extensively used to evaluate the efficacy of various *F. tularensis* vaccine candidates, likely has limitations due to extreme susceptibility of mice [22]. 

Previously, several research groups have utilized logistic modeling to perform multivariate analysis of the correlates identified using various types of in vivo and in vitro models, e.g., bacterial numbers, secreted cytokines, cytokine gene expression, lymphocyte stimulation indices, etc. [5,8,23,24,25]. For example, our group used human ex vivo data and identified the fewest number of data that with the highest accuracy predicted the immune status of a donor [25]. In another study, using mouse data, results were combined from in vivo gene expression data and a co-culture method and such strategy should be desirable, since the data likely will be complementary [23]. Similar analyses using data obtained from several sources, likely will enable comparisons between different types of tissues and species and such modeling should make it possible to identify putative correlates of protection. The correlates can then be validated in each of the relevant systems [26,27] and through this strategy, vaccine candidates can be assessed as a first step to achieve the requirements of the Animal Rule.

## 4. Materials and Methods 

### 4.1. Bacterial Strains

*F. tularensis* LVS was obtained from the American Type Culture Collection (ATCC 29684). *F. tularensis* subsp. *tularensis* strain SCHU S4 was obtained from the *Francisella* Strain Collection of the Swedish Defense Research Agency, Umeå, Sweden. The *clpB* deletion mutant of SCHU S4 (∆*clpB*) has been described previously [4]. The *clpB* and *wbtC* double deletion mutant of SCHU S4 (∆*clpB/*∆*wbtC*) was generated by introducing the *wbtC* deletion in the ∆*clpB* mutant using the allelic replacement method as previously described for ∆*clpB* [4]. The experimental work related to SCHU S4 was performed in a biosafety level 3 facility certified by the Swedish Work Environment Authority. 

### 4.2. Animals

In the co-culture experiments, the inbred female Fischer 344/IcoCrl rats used were obtained from Charles River, Italy. A dose of approximately 1 × 10^5^ CFU of the LVS or the ∆*clpB/*∆*wbtC* strain was used for SC immunization. The resulting infection gave no or very mild objective symptoms. The approval for the animal experiments was obtained from the Ethical Committee on Animal Research, Umeå, Sweden, A42/2016 and from ACURO CB-2016-16.05. For the rat challenge studies, female Fischer rats 344 rats were purchased from Harlan Laboratories (Indianapolis, IN). The work was approved by the UNMHSC Institutional Animal Use and Care Committee (Protocol 14-101234-HSC).

### 4.3. Immunization and Challenge of Fischer 344 Rats

A vial from each of the Δ*clpB*/Δ*wbtC* or the LVS vaccine stocks, stored at −80 °C in broth supplemented with 20% glycerol, was thawed and diluted in PBS to a final concentration of 5 × 10^7^ CFU/mL. An aliquot of each vaccine preparation was plated on selective cysteine heart agar plates (CHAB; Thermo Fisher Scientific Remel Products, Lenexa, KS) to verify the titer. For the SC immunization, the study animals were lightly anesthetized with isoflurane (Abbott Laboratories; Chicago, IL) and injected with 200 μL (1 × 10^7^ CFU/rat) SC in the interscapular area.

Four days before the challenge, a vial from the SCHU S4 working stock was thawed, plated onto CHAB plates, and cultured for 3 days at 37 °C. On the day before challenge, colonies were suspended into 4.5 mL Chamberlain’s Defined Medium (CDM) to produce a bacterial suspension with an optical density (OD) of 0.1 and 400 μL of the resulting bacterial suspension were inoculated into 100 mL of sterile CDM in a sterile 500 mL disposable Erlenmeyer flask with vented cap (Corning) and cultured at 37 °C for 17–20 h. The overnight culture was diluted to an OD of 0.1 with CDM, and 10 mL of nebulizer solution was prepared by diluting with Brain Heart Infusion Broth (BHIB; Teknova, Hollister, CA) to a specific CFU/mL based on the OD_600_ to achieve the target nebulizer concentration [28]. The nebulizer solutions were serially diluted and plated on CHAB plates to determine the actual pre-spray bacterial inoculum concentration.

Aerosolized organisms were generated using a Collison 3-jet nebulizer (BGI, Inc., Waltham, MA) and given to the study animals in a nose-only exposure chamber (In-Tox Products, Inc., Moriarty, NM). The generator flow rate was maintained at 7.5 L/min and the impinger flow rate was maintained at 5 L/min. In general, because the InTox aerosol exposure chamber can only accommodate up to 20 rats at a time, the animals in each study were divided across multiple aerosol exposure runs to avoid issues about run-to-run variability. In addition, an additional 1–3 animals were included in every aerosol run to directly measure lung deposition. The animals were exposed to aerosolized SCHU S4 for 15 min, and the system was purged for 2 min before the animals were removed. Particle sizes were measured 10 min into each exposure run by drawing an aerosol sample for 20 s. The aerosol sample was diluted 1:20 using an aerosol dilutor (TSI; Shoreview, MN) and analyzed using an aerodynamic particle sizer spectrometer (TSI). Samples from the inoculum in the generator were plated after each run onto CHAB plates to determine the actual challenge inoculum. The consistency of the delivered dose was also monitored by plating serial dilutions of samples collected directly from the aerosol exposure chamber into an all glass impinger (Ace Glass, Inc., Vineland, NJ, USA. The actual challenge dose was determined directly from the lung depositions obtained by sacrificing additional rats that were included in each exposure run and by plating serial dilutions of lung homogenates onto CHAB plates.

### 4.4. Generation of BMDM 

Bone marrow was obtained from femurs of Fischer 344 rats through flushing with Dulbecco’s Modified Eagle Medium (DMEM). After washing, a suspension was generated by resuspending cells in DMEM supplemented with 10% of heat-inactivated fetal bovine serum, 0.2 of mM L-glutamine, (Life Technologies) (sDMEM). After counting, bone marrow cells were suspended in fetal bovine serum (FBS) + 10% DMSO to a concentration of 1.0 × 10^8^ cells/mL and 1 mL aliquots were supplied per cryotube. The tubes were stored over night at −80 °C in a cryo-freezing container (Nalgene, Merck, Darmstadt, Germany) before being stored in liquid nitrogen until needed. One week before start of the experiment, the cells were thawed at 37 °C in a heating block and gently suspended in 37 °C warm sDMEM (9 mL per cryotube). The cells were collected by centrifugation (300 × *g* for 10 min) and the pelleted cells suspended in 10 mL sDMEM supplemented with 10% of L929 conditioned media and seeded into a 10 cm Petri dish that was incubated at 37 °C and 5% CO_2_. After 3 d, an additional 5 mL of sDMEM + 10% L929 conditioned media was added. After 7 d, medium was discarded and 10 mL of cold PBS with 10 mM EDTA was added and the cultures were incubated for 10 min on ice. The cells were centrifuged and suspended in sDMEM and 1 × 10^5^ cells were seeded per well in a 96-well plates (Falcon, Corning GmbH, Kaiserslautern, Germany) and incubated overnight at 37 °C and 5% CO_2_ and then used in the T-cell mediated intra-macrophage bacterial growth control assay (co-culture). The number of viable bone marrow-derived macrophages (BMDM) was determined after Trypan blue staining using a Vi-CELL XR cell viability analyzer (Beckman Coulter, Bromma, Sweden). 

### 4.5. Splenocyte Preparation

Splenocytes were prepared essentially as described [29]. Six to eight weeks following immunization, spleens were obtained, and a cell suspension was prepared by use of a plunger of a syringe. Then, the suspension was further purified by use of a cell strainer to prepare a single cell suspension. After centrifugation, ammonium chloride was added to lyse erythrocytes. The suspension was washed and resuspended in sDMEM. The viable splenocytes were enumerated after Trypan blue staining by counting in a Vi-CELL XR cell viability analyzer. Cells were either directly used in the co-culture assay or stored in liquid nitrogen. The co-culture assay is time-consuming if freshly prepared cells have to be used and therefore thawed splenocytes after storage at −80 °C was used in the assay. It was verified that the viability and performance of the splenocytes was similar to the viability of freshly prepared splenocytes. For storage in liquid nitrogen cells were suspended in FBS (5.0 × 10^8^) and incubated on ice for 10 min before DMSO was supplied (10% final concentration). Cryotubes were filled with 1 mL aliquots and stored overnight at 80 °C in a cryo-freezing container (Nalgene) before being stored in liquid nitrogen until needed. Three hours before onset of the co-culture assay the cells were thawed at 37 °C in a heating block and gently suspended in 37 °C warm sDMEM (9 mL per cryotube). The cells were collected by centrifugation (300 × *g* for 10 min) and the pelleted cells suspended in 10 mL of sDMEM and incubated at 37 °C 5% CO_2_ for 2 h. Thereafter the number of viable splenocytes was determined after Trypan blue staining using a Vi-CELL XR cell viability analyzer. 

### 4.6. Co-Culture Assay

SCHU S4 was cultivated overnight on Gc-agar plates and resuspended in sDMEM before addition to the BMDM monolayer. The cell cultures had been treated as described under “Generation of BMDM” and were infected at a multiplicity of infection (MOI) of 0.4 (bacteria:BMDM). After allowing for bacterial uptake for 2 h, medium was removed, and the cell monolayer was washed twice with PBS and 0.2 mL of sDMEM containing 20 µg/mL gentamicin was added to each well. Then, plates were incubated for 45 min and washed twice with PBS. The BMDM monolayers were overlaid with 0.2 mL of sDMEM with 5 × 10^5^ of congenic splenocytes, thus, a ratio of BMDM:splenocytes of 1:5. Bacterial numbers were determined by lysis and plating of serial dilutions of the lysates. In certain cultures, 1 mM of N^G^-monomethyl-L-arginine (NMMLA; MilliporeSigma) was added together with the splenocytes.

### 4.7. Nitrite Measurement 

The NO_2_^−^ concentration in the culture supernatants of the co-culture assay was determined as previously described [30]. Briefly, 50 µL of supernatant was mixed with 50 µL of the Griess reagents, p-Aminobenzenesulfonamide (58 mM in 5% H_3_PO_4_) and 2,6,8-Trihydroxypurine (3.9 mM) (Merck). After an incubation of 10 min, the A_540_ was recorded. A standard curve was created by use of sodium nitrite and the concentration of NO_2_^−^ determined. The lowest detectable concentration was 1.0 µM.

### 4.8. Multiplex Cytokine Analysis 

Cell culture supernatants, 50 μL/well, were collected from the wells of the co-culture assay after 48 h of incubation. The supernatants were sterile filtered and stored frozen at −80 °C until analyzed using a Bio-Plex 200 system (BioRad Laboratories Inc., Hercules, CA, USA) and either a commercial Rat 27-plex kit (MilliporeSigma) or a custom-made Rat 9-plex kit (MilliporeSigma) following the manufacturer’s instructions. The 9-plex kit contained the following cytokines: IL-2, IL-4, IL-12p70, IL-17A, IFN-γ, G-CSF, RANTES, fractalkine, and TNF. 

### 4.9. Flow Cytometry Analysis of Surface Markers and Intracellular Cytokine Staining 

After 48 h, cells were collected from the co-culture assay or after 72 h of incubation from the re-call stimulation assay. Non-adherent cells were collected and transferred to a new plate with the addition of 5 µg/mL of Brefeldin A (BD Biosciences, San Jose, CA, USA). Four hours later, plates were centrifuged and supernatants were removed. Cells were stained with Aqua Viability Dye (Molecular Probes/Invitrogen) in order to identify live and dead cells and thereafter labelled with conjugated monoclonal antibodies (mAb) recognizing intracellular cytokines or cell surface markers. The following mAb conjugates were used: CD3-Bv421 (clone 1F4, BD Biosciences), CD4-PeCy7 (clone OX-35, BD Biosciences) and CD8-Bv605 (clone OX-8, BD Biosciences), IFN-γ- eFluor660 (clone DB-1, eBiosciences), and TNF-PE (clone TN3-19.12, BD Biosciences). Cells were analyzed by use of an LSRII flow cytometer (BD Biosciences) and FACSDiva software (BD Biosciences, San Jose, CA, USA and FlowJo software (Tree Star, San Carlos, CA, USA). 

### 4.10. Data Analysis and Statistical Methods 

One way ANOVA with LSD post hoc test for multiple comparison was used to analyze the significance of differences between different treatment groups. A value of *p* < 0.05 was considered significant. The data were visualized using boxplots when multiple experiments formed the basis for the analysis. Correlations were estimated using Spearman’s correlation.

CFU, nitrite, and cytokine data were used to derive a classifier that enables prediction of treatment, i.e., to predict if the rat was immunized with any of the strains LVS, ∆*clpB*/∆*wbtC*, or not immunized at all (naïve). Linear discriminate analysis (LDA), using a classical setting assuming homoscedasticity and no prior, was used to build the classifiers and cross validation was used to predict the posterior probabilities. The cross-validation procedure, where part of the data is left out when building the model and instead used for independent testing, enable us to test the model’s ability to predict new blind data. The analyses were made using the functions discrim and crossvalidate in S-plus 8.2 (TIBCO Spotfire). The number of explanatory variables (nine cytokines, nitrite, and CFU) in the predictive models ranged from one to five and all possible models were considered, e.g., for models with two explanatory variables all 119 combinations of two unique variables were considered. For each model category the classifiers with the highest number of correctly classified rats were presented. 

## Figures and Tables

**Table 1:** Survival of *Francisella tularensis*-immunized Fischer rats after challenge with SCHU S4.

Exp.	Immunization	Immunization Dose	Challenge Dose	% Survival (n)	MTD (Day ± SEM)
1 ^a^	PBS	-	6.2 × 10^3^	0.0 (8)	4.5 ± 0.3
	LVS	9.0 × 10^6^	6.2 × 10^3^	50.0 (8)	7.0 ± 0.3 ^c^
	Δ*clpB*/Δ*wbtC*	1.0 × 10^7^	6.2 × 10^3^	37.5 (8)	7.3 ± 0.9 ^c^
2 ^a^	PBS	-	1.0 × 10^4^	0.0 (10)	4.4 ± 0.3
	LVS	1.4 × 10^7^	1.0 × 10^4^	13.0 (15)	5.9 ± 0.2 ^c^
	Δ*clpB*/Δ*wbtC*	9.8 × 10^6^	1.0 × 10^4^	0.0 (12)	5.8 ± 0.2 ^c^
3 ^b^	PBS	-	2.0 × 10^3^	0.0 (12)	4.1 ± 0.1
	LVS	1.8 × 10^7^	2.0 × 10^3^	41.7 (12)	6.4 ± 0.2 ^c^
	Δ*clpB*/Δ*wbtC*	1.1 × 10^6^	2.0 × 10^3^	16.7 (12)	6.5 ± 0.7 ^c^

^a^ Challenge of rats was performed 8 weeks after immunization. ^b^ Challenge of rats was performed 16 weeks after immunization. ^c^ Significantly delayed MTD (*p* < 0.05), compared to rats in the PBS-treated group.

**Table 2:** Correlation between cytokines and nitrite levels according to Spearman’s correlation test.

	Nitrite	IFN-γ	IL-2	Fractalkine	TNF	RANTES
Nitrite (µM)	-	0.71 **	0.76 **	0.71 **	0.73 **	0.66 **
IFN-γ	0.71 **	-	0.90 **	0.84 **	0.86 **	0.76 **
IL-2	0.76 **	0.90 **	-	0.92 **	0.94 **	0.83 **
Fractalkine	0.71 **	0.84 **	0.92 **	-	0.93 **	0.85 **
TNF	0.73 **	0.86 **	0.94 **	0.93 **	-	0.89 **
RANTES	0.66 **	0.76 **	0.83 **	0.85 **	0.89 **	-

** *p* < 0.01.

**Table 3:** Correlations between nitrite levels and cytokine levels in the respective co-culture group.

Cytokine	Nitrite ^1^		
	Naive	LVS	∆*clpB/*∆*wbtC*
IFN-γ	0.22 ^2^	0.74 **	0.44
IL-2	0.29	0.85 **	0.51 *
Fractalkine	0.43	0.68 **	0.50 *
TNF	0.26	0.76 **	0.49 *
RANTES	0.16	0.66 **	0.49 *

^1^ Nitrite levels from co-cultures with the indicated group of splenocytes were analyzed for their correlation to cytokine levels from the respective co-culture. ^2^ Spearman’s Rho. * *p* < 0.05. ** *p* < 0.01.

**Table 4:** Correlates of immunity against *F. tularensis* as suggested by the accuracy of prediction models.

Variables Included in the Model with the Highest Performance ^1^	Cross Validation ^2^
CFU	0.690
CFU + IFN-γ	0.931
CFU + IFN- γ + TNF	0.931
CFU + nitrite + IL-2 + TNF	0.948

^1^ See Section 4 for a description of the prediction models. ^2^ A cross validation value equal to 1.0 indicates that 100% of tested individuals are expected to be correctly classified.

**Table 5:** Assignment of rats to immunization group using linear discriminate analysis including the variables CFU, nitrite, IL-2, and TNF.

Predicted	Immunization ^1^		
	Naïve	LVS	Δ*clpB*/Δ*wbtC*
Naïve	21	0	2
LVS	0	18	0
Δ*clpB*/Δ*wbtC*	0	1	16

^1^ Rats were either naïve or immunized with LVS or ∆*clpB/*∆*wbtC.*

## Supplementary Materials

The following are available online at https://www.mdpi.com/2076-0817/9/5/338/s1, Table S1: Cytokine levels accumulated in the culture media of co-cultures after 48 h of incubation.
