# Supplementary material for: Vaccine-Mediated Mechanisms Controlling Francisella tularensis SCHU S4 Growth in a Rat Co-Culture System
Source: Pathogens. 2020 Apr 30;9(5):338. doi: 10.3390/pathogens9050338 (PMC7280961; doi:10.3390/pathogens9050338)
Supplement: Supplementary file 1 [file pathogens-09-00338-s001.pdf]

Table S1. Cytokine levels accumulated in the culture media of co-cultures after 48 h of incubation.

Cytokine/Chemokine	Naive	LVS-immune	Δ*clpB*/Δ*wbtC*-immune
G-CSF	−0.01 ± 0.07[a]	0.15 ± 0.04*[b]	0.07 ± 0.06
Eotaxin	0.48 ± 0.07	0.47 ± 0.11	0.58 ± 0.04
GM-CSF	1.35 ± 0.05	1.46 ± 0.05	1.56 ± 0.02*
IL-1α	1.15 ± 0.17	1.38 ± 0.13	1.38 ± 0.08
Leptin	1.64 ± 0.08	1.81 ± 0.03	1.73 ± 0.03
MIP-1α	2.44 ± 0.08	2.66 ± 0.08	2.68 ± 0.07
IL-4	0.12 ± 0.09	0.30 ± 0.10	0.27 ± 0.11
IL-1β	1.35 ± 0.19	1.53 ± 0.11	1.61 ± 0.13
IL-2	1.48 ± 0.06	2.77 ± 0.10*** (***)[c]	2.04 ± 0.18**
IL-6	1.45 ± 0.02	1.62 ± 0.16	1.48 ± 0.00
EGF	−1.29 ± 0.22	−1.08 ± 0.14	−1.07 ± 0.21
IL-13	−0.26 ± 0.00	−0.26 ± 0.00	−0.26 ± 0.00
IL-10	2.12 ± 0.13	2.08 ± 0.05	2.18 ± 0.02
IL-12p70	1.04 ± 0.11	0.96 ± 0.13	1.02 ± 0.12
IFN-γ	1.98 ± 0.05	3.61 ± 0.11*** (***)	2.65 ± 0.22**
IL-5	1.17 ± 0.08	1.44 ± 0.05*	1.41 ± 0.08*
IL-17α	1.25 ± 0.20	1.75 ± 0.12*	1.37 ± 0.20
IL-18	2.12 ± 0.05	2.09 ± 0.07	2.26 ± 0.07
MCP-1	2.98 ± 0.07	3.09 ± 0.10	3.14 ± 0.06
IP-10	3.27 ± 0.09	3.58 ± 0.10*	3.34 ± 0.05
GRO/KC	1.63 ± 0.08	1.30 ± 0.19	1.38 ± 0.24
VEGF	1.71 ± 0.21	1.64 ± 0.22	1.90 ± 0.12
Fractalkine	0.71 ± 0.04	1.14 ± 0.06*** (**)	0.81 ± 0.10
Lix	1.37 ± 0.13	1.49 ± 0.05	1.51 ± 0.01
MIP-2	2.05 ± 0.13	2.06 ± 0.11	2.15 ± 0.11
TNF	0.46 ± 0.07	1.19 ± 0.08*** (***)	0.64 ± 0.13
RANTES	1.97 ± 0.07	2.39 ± 0.06*** (*)	2.10 ± 0.09

[a] Mean ± standard error of mean. [b]Stars indicate significant difference *vs*. co-cultures with naive splenocytes, [c]Stars in brackets indicate significant difference *vs*. co-cultures with Δ*clpB*/Δ*wbtC*-immune splenocytes. * $P < 0.05$, ** $P < 0.01$, and *** $P < 0.001$.
